# Lemon Juice, Sesame Paste, and Autoclaving Influence Iron Bioavailability of Hummus: Assessment by an In Vitro Digestion/Caco-2 Cell Model

**DOI:** 10.3390/foods9040474

**Published:** 2020-04-10

**Authors:** Nour Doumani, Isabelle Severin, Laurence Dahbi, Elias Bou-Maroun, Maya Tueni, Nicolas Sok, Marie-Christine Chagnon, Jacqueline Maalouly, Philippe Cayot

**Affiliations:** 1 UMR PAM Food and Microbiological Processes, University of Burgundy Franche Comté/AgroSup Dijon, 1 esplanade Erasme, 21000 Dijon, France; elias.bou-maroun@agrosupdijon.fr (E.B.-M.); nicolas.sok@agrosupdijon.fr (N.S.); philippe.cayot@agrosupdijon.fr (P.C.); 2 Department of Chemistry and Biochemistry, Faculty of Sciences II, Lebanese University, Jdeideth El Matn, Fanar 90656, Lebanon; j_maalouly@hotmail.com; 3 Department of Biology and Nutrition, Faculty of Sciences II, Lebanese University, Jdeideth El Matn, Fanar 90656, Lebanon; mayatueni@hotmail.com; 4 NUTOX UMR INSERM 1231 Laboratory of Nutrition, Physiology, and Toxicology, University of Burgundy Franche Comté/AgroSup Dijon, 1 esplanade Erasme, 21000 Dijon, France; isabelle.severin@agrosupdijon.fr (I.S.); laurence.dahbi@u-bourgogne.fr (L.D.); marie-christine.chagnon@agrosupdijon.fr (M.-C.C.)

**Keywords:** non-heme iron, plant-based food, Mediterranean and middle-eastern cuisine, hummus, food processing and formulation, in vitro digestion, iron dialysis, iron uptake by Caco-2 cells

## Abstract

Hummus, an iron-containing plant-based dish mainly made from chickpea purée, tahini, lemon juice and garlic, could be a valuable source of iron when bioavailable. Since the processing and formulation of food influence iron bioavailability, the present study investigated for the first time, their effects on hummus. Firstly, iron bioaccessibility was assessed on eight samples (prepared according to the screening Hadamard matrix) by in vitro digestion preceding iron dialysis. Then, iron bioavailability of four selected samples was estimated by the in vitro digestion/Caco-2 cell model. Total and dialyzable iron were determined by the atomic absorption spectrometry and ferritin formation was determined using an ELISA kit. Only autoclaving, among other processes, had a significant effect on iron bioaccessibility (+9.5, *p* < 0.05). Lemon juice had the highest positive effect (+15.9, *p* < 0.05). Consequently, the effect of its acidic components were investigated based on a full factorial 2^3^ experimental design; no significant difference was detected. Garlic’s effect was not significant, but tahini’s effect was negative (−8.9, *p* < 0.05). Despite the latter, hummus had a higher iron bioavailability than only cooked chickpeas (30.4 and 7.23 ng ferritin/mg protein, respectively). In conclusion, hummus may be a promising source of iron; further in vivo studies are needed for confirmation.

## 1. Introduction

Iron is an essential element for the well-functioning of the human body. It plays a vital role in the formation of hemoglobin, the substance in red blood cells that carries and delivers oxygen from the lungs to the organs [1]. A lack of iron causes the dysregulation of oxygen transport, thereby leading to health problems such as anemia, cognitive impairment, and low work productivity [2,3,4]. Iron deficiency is most prevalent among the group of individuals with increased body needs of iron, such as children for growth and development, and pre-menopausal women during menstruation, pregnancy and lactation [5]. 

A common reason for iron deficiency is the inadequate dietary intake of iron. Dietary iron exists in two forms: heme iron and non-heme iron [6]. Heme iron is the most easily absorbed form of iron, as it is bound to a protein and is thought to be transported directly inside the enterocytes by a specific heme transporter [7]. In contrast, non-heme iron which is the ionic form of iron (usually Fe^3+^ in food) is less-well absorbed due to the presence of potent inhibitors of iron absorption such as phytic acid, polyphenol, and tannins. These inhibitors, known as antinutritional factors, are plant compounds that reduce the body’s ability to absorb the essential nutrients [8].

While heme iron is more absorbed than non-heme iron, it is only found in animal products. In addition to supplementation, biofortification and fortification strategies, animal product consumption can help alleviate nutritional iron deficiency. Nevertheless, alternative strategies for animal consumption must be implemented taking into consideration vegetarians and vegans, individuals with financial problems, and the increasing threat of farming and animal production on the environment [9,10]. The United Nations Environment Program (UNEP) in 2010 [11] called for shifting to alternative sources of animal protein such as plant-based products. Plants such as legumes are good sources of protein and are also considered to have a high content of iron [12] whether well or poorly available for absorption. 

Hummus is a dish based on a purée of cooked chickpea, commonly consumed in Mediterranean and Middle Eastern countries [13,14]. It has also become popular worldwide and is commercialized in the European markets [15], the United Kingdom [16,17], and the United States [14]. The consumption of hummus has grown enormously recently, and the global hummus market size was reported by the Market Research Future to exhibit a compound annual growth rate (CAGR) of 12.84% during the forecast period from 2018 to 2027 [18]. It has gained attention, not only for having a special flavor and varied consumption forms but also for fitting in the spectrum of the recent preferences of the consumers of plant-based proteins (alternative protein) [15], as well as having a high nutrient profile [19]. Hummus is rich in proteins, vitamins, and iron (2.44 mg/100 g) among other minerals [13,14,19].

Iron in hummus, like in other plant-based products, is the non-heme iron form that is poorly absorbed. Several factors influence iron absorption, namely: the iron status of the consumer, the dietary factors, as well as the processing and formulation of the food [20]. The latter can improve iron assimilation in humans by increasing or conserving the content of enhancers such as ascorbic acid and decreasing the content of inhibitors such as phytic acid [21,22,23]. The relative proportions of enhancers and inhibitors are important to be identified in a non-heme iron-containing meal, as they affect the amount of iron absorbed [24].

The preparation of hummus consists of the processing of chickpeas, and the addition of tahini, lemon juice, and garlic as the main ingredients [25]. The usual processing of chickpeas to make hummus includes soaking, cooking, and sometimes the removal of the seed coat [25]. The literature has shown that soaking legumes decreased the antinutritional factors (e.g., phytate, tannins, oligosaccharides, and phenolic compounds) especially when bicarbonate was added [26,27]. Germination, though uncommon for hummus preparation [25], was shown to decrease the antinutritional factors in legumes more efficiently than soaking [23]. Cooking chickpeas using a pressure cooker was shown to be more effective in reducing the content of iron absorption inhibitors (phytic acid and tannins) than boiling [23,28,29,30]. This could be due to the higher temperature in the autoclave resulting in a shorter cooking duration. The latter, unlike high pressure, has been shown in a recent study to be well correlated with iron bioavailability [31]. The decortication of chickpeas decreased the antinutritional factors content and improved iron bioavailability [32]. Regarding the ingredients, lemon juice was shown to increase iron bioaccessibility and bioavailability from different meals (e.g., rice and pork meat) [33,34], garlic was shown to increase iron bioaccessibility in legumes [35,36] and tahini was suspected to decrease it [37]. The majority of the studies involving chickpea did not investigate chickpea as a component of a whole complex dish, nor assessed several of the above-cited factors combined.

Therefore, the aim of this study was to test the effect of processing (i.e., chickpea processing) and formulation (i.e., the addition of ingredients) on iron absorption from hummus. Seven factors were of interest: soaking/germination, bicarbonate addition, cooking by boiling/autoclaving, seed coat removal, the addition of tahini, lemon juice, and garlic. 

Clinical studies involving human subjects are the most accurate methodology to assess iron bioavailability from food. However, their feasibility is restricted by ethical, financial and duration limitations. Other alternative techniques have been used to estimate iron bioaccessibility and bioavailability: the in vitro digestion preceding the iron dialysis, and the in vitro digestion coupled to the iron uptake by the Caco-2 cells [38]. The in vitro digestion/Caco-2 cell model was developed more than twenty years ago [39,40,41,42,43] and has gained much attention as a method for iron bioavailability prediction in the last decade [39,44,45,46,47]. It was used to predict iron bioavailability from different food products, staple food crops, and forms of iron [48]. In addition, it has been shown to correlate with human studies [49]. Although it is more cost-effective and time-saving than clinical studies [49], it remains a delicate and sophisticated in vitro method requiring special cells care in comparison to other in vitro studies. Therefore, we used the in vitro digestion preceding the iron dialysis as a first screening tool for choosing the samples of interest, and then we further investigated them by exposure to the Caco-2 cells.

In this article, two terms will be widely used: “iron bioaccessibility” and “iron bioavailability”. By definition, “iron bioaccessibility” is the amount of ingested iron that is potentially available for absorption; it is dependent only on its digestion and release from the food matrix. Whereas “iron bioavailability” is defined as the amount of ingested iron that is available for absorption, absorbed by the enterocytes, and utilized in physiological functions. It is dependent on its digestion, release from the food matrix, absorption by intestinal cells, and transport to body cells [38]. This means that for a nutrient to be bioavailable, it should be bioaccessible first [50]. However, a bioaccessible nutrient is not definitely bioavailable but only potentially ready for absorption [51]. Both iron bioaccessibility and bioavailability will be estimated in this study as they are not directly measured in humans, in whom a complexity of influencing factors exists (e.g., dietary, luminal, and systemic factors) [52].

This study had three main objectives: the first objective was to assess the bioaccessibility of iron from eight hummus recipes processed and formulated differently, as well as the effect of each factor on iron bioaccessibility. Secondly, the samples of interest from the previous experiment were selected and further assessed for their iron bioavailability. Finally, the major components (citric, malic and ascorbic acid) of the factor with the largest effect on iron assimilation (lemon juice) were investigated for their effects on iron bioaccessibility from hummus. Throughout the study, iron bioaccessibility was estimated using the in vitro digestion preceding the iron dialysis through a semi-permeable membrane, and iron bioavailability was predicted using the in vitro digestion/Caco-2 cells model. The existing relationship between these two techniques was investigated as well. 

## 2. Materials and Methods 

### 2.1. Materials 

#### 2.1.1. Hummus Preparation and Iron Determination

The classical ingredients of hummus are processed chickpeas, tahini (sesame paste), lemon juice and garlic [25]. The type of chickpeas used were Kabuli from the most sold brand, Mexican La Macarena in the Lebanese market. The tahini used in this study was from a famous Lebanese brand, Wadi el Akhdar. Lemon juice and garlic were purchased from the local market. To test the effect of acids on iron bioaccessibility, DL-Malic acid, citric acid and L-ascorbic acid from Sigma Aldrich were used. Ferrous sulfate heptahydrate (RP Normapur; Prolabo, Paris, France) and a hydrochloric acid solution ≥37% (Sigma-Aldrich, Steinheim, Germany) were used for the standard solutions preparation and iron determination. Syringes and syringe micro-filters, used to filter the buffer solution containing the dialyzable iron, were purchased from Corning Incorporated, New York, NY, USA.

#### 2.1.2. In Vitro Digestion

The gastric digestion was obtained using pepsin from porcine gastric mucosa ≥250 units/mg (Sigma #P7000), and the intestinal digestion was obtained using pancreatin from porcine pancreas 4USP (Sigma #P1750), bile extract porcine (Sigma #B8631), and a 10 KDa dialysis membrane (SnakeSkin^®^ Dialysis Tubing, Thermo Scientific, Paisely, UK). Sodium bicarbonate was purchased from Normapur Prolabo, France. Sodium hydroxide, hydrochloric acid 6 M, potassium chloride and sodium chloride were used in the digestion and were purchased from Sigma Aldrich. 

#### 2.1.3. Cell Growth and Maintenance

Dulbecco’s modified Eagle’s medium (DMEM) high glucose (PAN BioTech, Aidenbach, Germany), fetal bovine serum (Dutscher), L-glutamine solution 200 mM (Gibco, Thermo Fisher Scientific, Paisely, UK), antibiotic-antimycotic solution 100× (Gibco, Thermo Fisher Scientific, Paisely, UK), minimal essential medium non-essential amino acids solution 100× (MEM NEAA; Gibco, Thermo Fisher Scientific, Paisely, UK), 4-(2-hydroxyethyl)-1-piperazine-ethane-sulfonic acid (HEPES; Sigma #H3375) were purchased and used for Caco-2 cell growth and maintenance.

#### 2.1.4. Iron Uptake by Caco-2 Cells Experiment

Eagle’s minimum essential medium (MEM; Gibco, Thermo Fisher Scientific, Paisely, UK), sodium bicarbonate solution (NaHCO_3_) for cell culture 7.5% (Gibco, Thermo Fisher Scientific, Paisely, UK) that has a pKa between 6.3 and 10.3, meaning a buffer zone between 5 and 7 and another one between 9.6 and 11, piperazine-N,N′-bis(2-ethanesulfonic) acid buffer solution pH 6.8 (PIPES at 1 mol/L; VWR international), human epidermal growth factor 2 mg (hEGF; Sigma #E9644), D-(+)-glucose solution (45%) for cell culture (Sigma), hydrocortisone 1 g (Sigma #H0888), insulin from bovine pancreas 50 mg (Sigma #I6634), antibiotic-antimycotic solution 100× (Gibco, Thermo Fisher Scientific, Paisely, UK), 3,3′,5-triiodo-L-thyronine 250 mg (Sigma #T2877), sodium selenite (Na_2_SeO_3_) suitable for cell culture ≥98% (BioReagent, Sigma), 6-well plates treated with collagen (Corning Biocoat, Corning, NY, USA), 15 KDa molecular weight cut-off dialysis membrane (Spectra/Por7 Dialysis Membrane, Spectrum Laboratories, Rancho Dominguez, CA, USA), Transwell inserts support for 6-well plate (Falcon) and silicone O-ring (40 mm, Lyreco) were all purchased.

#### 2.1.5. Ferritin and Protein Determination from Caco-2 Cells

PBS phosphate-buffered saline (PBS) solution for cell culture pH 7.4 (Gibco, Thermo Fisher Scientific), 200 µL Cellytic M (Sigma C2978), Human Ferritin ELISA Kit (Thermo Scientific), Coomassie Plus™ protein assay reagent (Thermo Scientific), 96-well microplates for protein determination (Greiner bio-one, Frickenhausen, Germany) were all purchased.

The Caco-2 cell line was provided by Sigma at passage 54 and stored in liquid nitrogen.

### 2.2. Methods

#### 2.2.1. Preparation of the Eight Samples of Hummus with Different Processing and Formulation (HDPF_I->VIII_) 

The preparation of hummus was close to the habits of hummus preparation in Lebanon [25], involving the same kind of processes and the same usual ingredients such as chickpeas, tahini (sesame paste), lemon juice and garlic. This work aimed to study the impact of seven factors of processing and formulation on iron bioaccessibility in hummus. The Hadamard matrix (7 factors 8 experiments, circular permutation by the right), a screening tool that permits the study of the effects of the 7 factors, was employed. Eight different preparations were obtained (Table 1). The preparation of the eight samples was divided into two sections: the processing of chickpeas, and the formulation of hummus. The processing steps included soaking or germination, without or with bicarbonate, boiling or autoclaving, conservation or removal of the seed coat of the chickpeas. All the chickpeas were soaked in distilled water in petri dishes layered with a papier Joseph (porous paper) under permanent lighting and at room temperature, with bicarbonate (0.1%) or without it. The chickpeas for soaking only were removed after 12 h. The chickpeas for germination were kept for an additional 48 h and covered with a perforated aluminum foil under the same lighting and temperature conditions. The chickpeas undergoing germination were rinsed with distilled water three times per day and sprinkled with water. As for the cooking of chickpeas, the boiling of chickpeas took place in distilled water in a pneumatic glass trough for 90 min, and the autoclaving was done using the Prestige Medical Classic Autoclave (210004 UK) for 20 min at 121 °C. When needed, the seed coats were removed manually while wearing gloves.

The formulation included the addition or not of: tahini (sesame paste), lemon juice and/or garlic to the cooked chickpeas. The quantities of the selected ingredients (Table 1) were based on 25 g dry chickpeas and were chosen according to famous Lebanese recipes [53,54,55]. The tahini was added from the tahini container conserved at 5 °C, lemon juice was squeezed directly from the lemon, and the garlic was peeled, cut into pieces and added to the chickpeas. The constituents of the eight samples were toughly mixed and homogenized using a mixer and grinder (Moulinex mini hachoir ilico white DJ200031). After the preparation of the eight samples, they were freeze-dried and then conserved at −20 °C forming lyophilized powdered samples.

#### 2.2.2. Preparation of the Eight Samples of Hummus with Different Acids (HDA_1->8_)

To test the effect of the basic acid components of lemon juice on iron bioaccessibility in hummus, a classical hummus formulation without lemon juice was prepared. The acidic components of lemon juice (citric, malic, and ascorbic) were instead added in different combinations according to the 2^3^ full factorial experimental design (Table 2). The classical hummus formulation consisted of cooked chickpeas (73%), tahini (26%) and garlic (1%). The effects of the acids (citric, malic and ascorbic) on iron dialyzability were studied at 4 mmol/100 g hummus, each. The amount and nature of the added acids differed: the acids were all absent (HDA_1_), added alone (HDA_2_, HDA_3_, and HDA_5_), added in dual (HDA4, HDA_6_, and HDA_7_) or in triple combinations (HDA_8_). 

#### 2.2.3. In Vitro Digestion and Iron Dialysis through the Dialysis Membrane

The samples HDPF_I->VIII_ (hummus with different processing and formulation) and HDA_1->8_ (hummus with different acids) were digested in vitro following the procedure described by Chiochetti et al. (2018) [56] with some modifications. The in vitro digestion consisted of two major steps consisting of gastric digestion and intestinal digestion. The gastric digestion started with the homogenizing of 3 g of each freeze-dried hummus sample with 20 mL of distilled water. Afterward, the pH was adjusted to 2, and the pepsin enzyme was added to obtain 2000 U·mL^−1^ in the final digestion mixture [57]. The digesta was bubbled with argon (mimicking the anaerobic environment of the intestines, by the escape of oxygen), and then kept in the water shaking bath at 37 °C with 100 oscillations per minute. After 1 h of gastric digestion, the pH of the sample was adjusted to 7, then 10 g of the digesta were transferred to a 10 KDa dialysis membrane submerged in a buffer solution (0.1 M NaHCO_3_) for half an hour in the water shaking bath at 37 °C with 100 oscillations per minute. Meanwhile, a pancreatin–bile solution was prepared by mixing pancreatin (2 mg/mL) and bile extract (12 mg/mL) in a 0.1 M NaHCO_3_ solution. Afterward, 5 mL of the freshly prepared pancreatin–bile solution was added, then the pH was readjusted to 7, and the sample was shaken for an additional 2 h in the same conditions, to achieve the intestinal phase and the dialysis of iron (see Supplementary Information: Figure S1). The dialysis membrane was removed and the buffer solution of each sample was filtered and conserved at −20 °C until dialyzable iron determination. All the glassware was cleaned in distilled water, soaked in 2% (*v*/*v*) HCl solution overnight and then rinsed with distilled water before use throughout the experiment. 

#### 2.2.4. Determination of the Total and Dialyzable Iron in the Samples

For the determination of the total iron content in the freeze-dried HDPF and HDA samples, the samples were firstly dried in an oven (102 °C) and then dry-ashed in a furnace (Nabertherm) at 550 °C for 16 h. Finally, a clear solution of ash dissolved in 2% (*v*/*v*) HCl was prepared and used for the determination of the total iron by the atomic absorption spectrophotometer. As for the determination of the dialyzable iron from the “digested samples”, the buffer solution containing the dialyzable iron was filtered and then used directly for the determination of the dialyzable iron by the atomic absorption spectrometer. The atomic absorption spectrometer (SpectrAA 220, Varian, Australia) was used along with the iron lamp (5 mA; linearity range: 0.06–15 ppm) and followed a radiation of 248.3 nm. A standard curve was prepared by analyzing solutions containing known iron concentrations (0.05–1 ppm of ferrous sulfate and r^2^ = 0.996) using the Beer–Lambert law. Each measurement was an average of three acquisitions.

#### 2.2.5. Cell Culture

The Caco-2 cells stored in liquid nitrogen were used for the iron uptake experiment. Before their use in the uptake study, they were grown in a T75 culture flask with a high glucose DMEM (Dulbecco’s modified Eagle’s medium) supplemented medium. The medium also consisted of 10% (*v*/*v*) of fetal bovine serum, 1% (*v*/*v*) of MEM non-essential amino acids solution, 25 mM (*m*/*v*) HEPES solution (pH 7.4), 1% (*v*/*v*) of 4 mM l-glutamine solution and 1% (*v*/*v*) of an antibiotic-antimycotic solution. The cells were also maintained by changing the medium every 48 h, in an incubator at 37 °C at a constant humidity and in 5% CO_2_ and 95% air atmosphere.

Caco-2 cells between passages 69–70 were seeded into 6-well plates treated with collagen (Corning BioCoat, USA) at a density of 4.75 × 10^−5^ cells/well in 2 mL of the supplemented DMEM. The medium was changed every 48 h for a 13–15 days’ period according to the technique implemented by Glahn et al. (1998) [40]. After 13–15 days, Caco-2 cells became ready to be used in the iron uptake experiment. Twenty-four hours before the experiment, the 2 mL supplemented DMEM medium were replaced with a 2 mL supplemented MEM (Eagle’s minimum essential medium) without the fetal bovine serum. The supplements were a mixture of nine elements according to Glahn et al. (1998). Two buffers for media regulation were used: NaHCO_3_ (26.1 mM), and PIPES (10 mmol/L). Seven elements for cell proliferation were added: epidermal growth factor (20 µg/L), glucose (19.4 mmol/L), hydrocortisone (11 µmol/L), insulin (0.87 µmol/L), triiodothyronine (0.05 µmol/L), Na_2_SeO_3_ (0.02 µmol/L) and an antibiotic-antimycotic solution (1%). On the day of the experiment, the 2 mL of the supplemented MEM medium were replaced with 1 mL for only 1 h during the second part of the intestinal digestion.

#### 2.2.6. In Vitro Digestion of the Four Hummus Samples and Iron Uptake by the Caco-2 Cells 

The hummus samples were digested in vitro following the procedure described by Glahn et al. (1998) with some modifications [40]. The in vitro digestion consisted of three major steps: (a) the gastric digestion, (b) the 1st part of the intestinal digestion, and (c) the 2nd part of the intestinal digestion, which is the uptake phase where the cells are exposed to the supernatant containing the intestinal digesta. The gastric digestion started with homogenizing 3 g of each freeze-dried sample with 25 mL of saline solution (140 mmol of NaCl and 5 mmol of KCl) at pH 2. Then the pepsin enzyme was added to obtain 2000 U·mL^-1^ in the final digestion mixture [57]. The digesta was bubbled with argon to make sure oxygen was not present for anaerobic conditions and then incubated in the water shaking bath at 37 °C with 100 oscillations per minute. After 1 h of gastric digestion, the pH of the sample was gradually adjusted to pH 5.5 with a 1 M NaHCO_3_ solution. Meanwhile, a pancreatin–bile solution was prepared by mixing pancreatin (2 mg/mL) and bile extract (12 mg/mL) in a 0.1 M NaHCO_3_ solution. Afterwards, 5 mL of the freshly prepared pancreatin–bile solution was added, then the pH was readjusted to 7, and the digesta was incubated in the water shaking bath at 37 °C with 100 oscillations per minute for 1 h to simulate the first part of the intestinal digestion. After 1 h, the digesta was centrifuged at 550× *g* for 15 min and the supernatant was used for the iron uptake experiment. The supernatant (1.5 mL) was added to an upper chamber above the cells. The upper chamber was created exclusively for this experiment by fitting an appropriately sized Transwell insert shortened at the bottom by 1 mm [56] and attached to a 15 KDa molecular weight cut-off dialysis membrane (Spectra/Por7 dialysis tubing, Spectrum laboratories, Europe) with a silicone O-ring (see Supplementary Information: Figure S2). The semi-permeable dialysis membrane allowed iron to diffuse into the lower chamber’s medium where the Caco-2 cells were bathed. The supernatants—simulated digestions of hummus—were kept for one hour above the cells on a shaking plate (Titramax 100, speed 3) inside the incubator at 37 °C, with constant humidity and in a 5% CO_2_ and 95% air atmosphere. After 1 h of incubation, the upper chamber with its content was removed. One milliliter of the supplemented MEM was added and the cells were incubated for an additional 23 h to allow the ferritin formation. 

#### 2.2.7. Cell Harvest

After incubating the cells for 23 h, the medium was removed. Afterward, the cells were rinsed twice with cold PBS (phosphate buffer saline) and harvested by adding 200 µL Cellytic M (Sigma) and by scraping them. The lysed cells were aspirated into micro tubes forming pellets, and they were kept for 15 min in ice then conserved at −80 °C until ferritin and protein determination. 

#### 2.2.8. Ferritin and Protein Determination

For ferritin and protein determination, the cells were thawed and then centrifuged at 13,000× *g* for 15 min at 4 °C to discard the cellular debris from the proteins contained in the supernatant. The ferritin content was determined by the Human Ferritin ELISA Kit (Thermo Scientific) and was measured using a microplate reader (infinite 200 pro Tecan i-control, Switzerland) at 450 nm with a reference wavelength set at 550 nm according to the kit instructions. The Coomassie Plus Assay Kit (Thermo Scientific) was used to determine the total protein content at 595 nm by the microplate reader. The determination of the latter was necessary to normalize the ferritin concentration. 

#### 2.2.9. Definition of Total Iron, Dialyzable Iron, Percentages of Bioaccessible Iron and Bioavailable Iron

The total iron content is the total amount of iron in the samples before the digestion. The dialyzable iron is the fraction of iron that crosses the dialysis membrane at the end of the in vitro digestion. The percentage of accessible iron is estimated by the following formula [58]:

(1) $$\frac{\mathrm{Dialyzable}\;\mathrm{iron}}{\mathrm{Total}\;\mathrm{iron}}\times 100,$$

Ferritin formation in Caco-2 cells expressed as the [content of ferritin in ng]/[total protein content in mg] is a marker of cell iron uptake [42]; it is analyzed to determine whether the endogenous iron released by the hummus samples could be bioavailable to intestinal cells. Throughout the manuscript, the “bioaccessibility” and “bioavailability” of iron were estimated by the measurements of its dialysis and the ferritin formation by the Caco-2 cells, respectively, after the in vitro digestion.

#### 2.2.10. Statistical Analysis

The data set is presented as mean values with the standard error of the mean (SEM) or the interval of confidence (IC) at 95% level. The statistical analysis for multiple comparisons was obtained by using the one-way ANOVA test followed by Tukey’s post-hoc test at 95% confidence. The analysis of the factors’ effects on iron bioaccessibility, Pearson’s correlation, and all the above-cited analyses were done by using the Minitab^®^ 18. The statistical analysis of the ferritin formation data was performed using the GraphPad Prism 8 software package.

## 3. Results

### 3.1. Amount of the Total Iron, the Dialyzable Iron, and the Percentage of Bioaccessible Iron in the HDPF Samples

**Figure 1:**
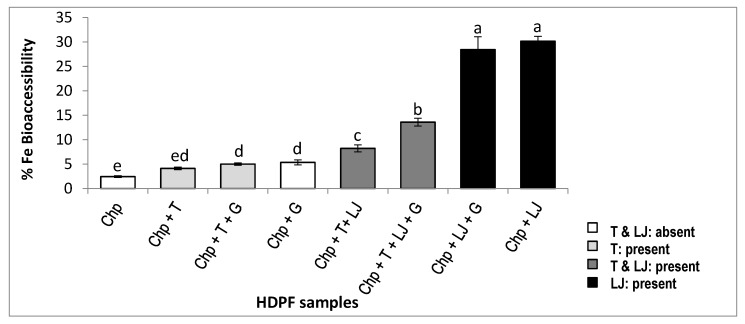
Percentage of iron bioaccessibility (dialyzable iron content in mg/100 g divided by the total iron content in mg/100 g) in the eight samples of HDPF chosen based on the Hadamard matrix, circular permutation by the right. (HDPF: hummus with different processing and formulation). Results are expressed as a mean ± IC (*n* = 3; IC 95%). Bars not sharing a letter are significantly different (*p* < 0.05), according to the one-way ANOVA test followed by Tukey’s post-hoc test at 95% confidence. Chp: chickpea, T: tahini, LJ: lemon juice, G: garlic. Table 1 provides further information about the processing of chickpeas.

Table 3 shows the amount of the total iron, the dialyzable iron and the iron bioaccessibility percentage in the freeze-dried samples of HDPF_I→VIII_ (hummus with different processing and formulation). The percentage of iron bioaccessibility is also illustrated in Figure 1.

The total iron content is similar in all the HDPF samples (an average of 4.7 mg/100 mg). The iron bioaccessibility differs among the samples according to the processing and formulation of hummus. The highest percentage is observed for the samples containing the lemon juice but not the tahini: samples II (30.17%) and VI (28.44%). These two samples have autoclaved chickpeas and lemon juice in common and they do not contain tahini. However, when tahini is present alongside boiled chickpeas and lemon juice, the iron bioaccessibility decreases by 72% for sample IV. The decrease is less pronounced for sample V, which also contains garlic, showing thereby an enhancing effect of garlic. Iron bioaccessibility percentage is the lowest where tahini is present and lemon juice is absent. Lemon juice might have an important effect on the dialysis of iron. Garlic might also have an enhancing effect. On the contrary, the observation suggests that tahini is an inhibitor of the dialysis of iron. The effects of the processing of chickpeas cannot be only analyzed from the above table; a Pareto chart is inevitable to confirm these results.

### 3.2. Effects of the Processing and Formulation Factors on Iron Absorption from Hummus 

**Figure 2:**
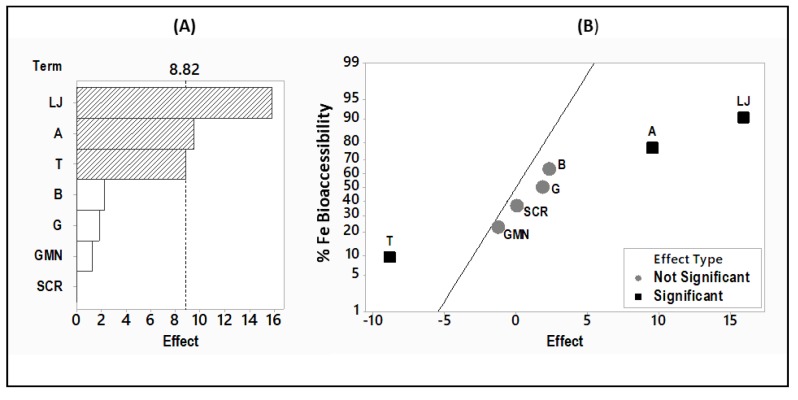
Pareto chart (**A**) and the Normal plot (**B**) of the effects of the seven factors of processing and formulation on iron bioaccessibility in the HDPF samples according to the Hadamard matrix, circular permutation by the right (α = 0.05). (HDPF: hummus with different processing and formulation). Chp: chickpea, T: tahini, LJ: lemon juice, G: Garlic, A: autoclave, B: bicarbonate, GMN: germination, SCR: seed coat removal.

For a finer analysis, the effect of each factor was tested individually following the Hadamard matrix, circular permutation by the right. The results are illustrated by a Pareto chart and a Normal plot of the standardized effects (Figure 2A,B, respectively). 

In the Pareto chart (Figure 2A) the standardized effects are presented from the largest effect to the smallest effect. The bars that cross the reference line are statistically significant. The bars of the following factors: LJ (lemon juice), A (autoclave), and T (tahini) cross the reference line (8.82) with the largest effect for the lemon juice. These factors are statistically significant at the 0.05 level. While the Pareto chart shows the absolute value of the effects, the Normal plot of the standardized effects (Figure 2B) reveals the magnitude and direction of the effects on iron bioaccessibility percentage: lemon juice and autoclave have a significant positive effect (+15.88 and +9.52 respectively with *p* < 0.05), whereas tahini has a significant negative effect (−8.87 with *p* < 0.05) on iron bioaccessibility. As for the other factors: soaking/germination, bicarbonate addition, seed coat removal and garlic addition, their effect on iron bioaccessibility from hummus is not significant, as they do not cross the reference line (+8.82).

### 3.3. Validation with Ferritin Uptake by Caco-2 Cells 

**Figure 3:**
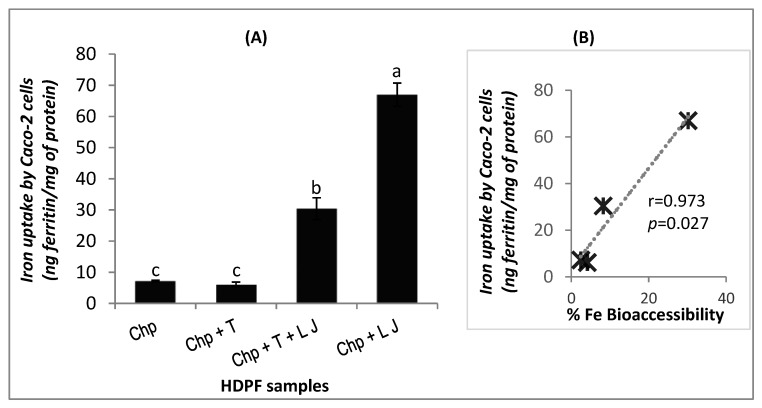
(**A**) Iron uptake (expressed as ng of ferritin/mg of total protein content in Caco-2 cells) from four HDPF samples (HDPF_VIII, I, II, and IV_). (**B**) Pearson’s correlation test between iron bioaccessibility (%) and iron uptake (ng ferritin/mg of protein) from the four HDPF samples. (HDPF: hummus with different processing and formulation). Results are expressed as a mean ± IC (*n* = 7; IC 95%). Bars not sharing a letter are significantly different (*p* < 0.05), according to the one-way ANOVA test followed by Tukey’s post-hoc test at 95% confidence. Chp: chickpea, T: tahini, LJ: lemon juice, G: garlic. Table 1 provides further information about the processing of chickpeas.

Lemon juice, autoclaving and tahini are the factors shown to have a significant effect on iron bioaccessibility from hummus. However, from the three cited factors, only lemon juice and tahini are essential for the preparation of hummus. Therefore, the effect of lemon juice and tahini on iron bioavailability were of higher interest for investigation. The samples: chickpea alone (VIII), chickpea with tahini (I) or lemon juice (II) or their combination (IV) previously prepared, were digested again and fed to Caco-2 cells. Ferritin formation is a marker of the iron uptake by the cells, which predicts iron bioavailability and is shown in Figure 3A. 

Figure 3A shows a similar trend of iron bioavailability from samples: I, II, IV, and VIII in comparison with the results of iron bioaccessibility from Figure 1; the iron bioavailability from chickpeas only, is 7.23 ng ferritin/ mg protein and shows a similar result when tahini is present along with chickpeas (6.14 ng ferritin/ mg protein, with *p* > 0.05). On the contrary, the addition of lemon juice to the chickpeas increases iron bioavailability greatly reaching its highest value (67 ng ferritin/ mg protein, with *p* < 0.05). When tahini and lemon juice are both added to chickpeas, iron bioavailability is reported to be 30.4 ng ferritin/ mg protein, which is approximately the mean of the iron bioavailability values of chickpeas and tahini on one hand, and chickpeas and lemon juice on another hand. As these results are very similar to the trend obtained with iron bioaccessibility (Figure 1), a Person’s correlation test was done to investigate if a linear association between iron bioaccessibility and iron bioavailability percentages, exists. The result of the test (Figure 3B) shows a significant positive linear correlation between iron bioaccessibility and iron bioavailability: the correlation coefficient is equal to 0.973 and is larger than the critical value (0.950) for a level of significance ≤0.05 [59] (see Supplementary Information: Figure S3).

### 3.4. Amount of Total Iron, Dialyzable Iron, and Percentage Accessible Iron in the HDA Samples

**Figure 4:**
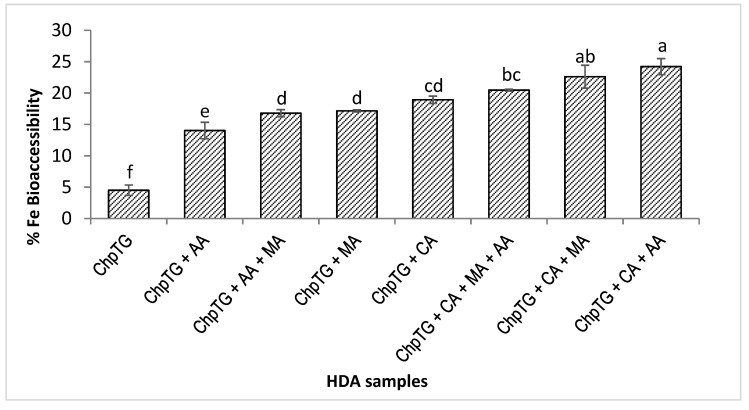
Percentage of iron bioaccessibility (dialyzable iron content (mg/100 g) divided by the total iron content (mg/100 g)) in the eight samples of HDA chosen based on the 2^3^ experimental design (full factorial). (HDA: hummus with different acids). Results are expressed as a mean ± IC (*n* = 3; IC 95%). Bars not sharing a letter are significantly different (*p* < 0.05), according to the one-way ANOVA test followed by Tukey’s post-hoc test at 95% confidence. ChpTG = Chp: chickpea + T: tahini + G: garlic. CA: citric acid, MA: malic acid, AA: ascorbic acid. Each acid is at 4 mmol/100 g of ChpTG.

As the highest estimated iron bioaccessibility and bioavailability of iron was obtained with the lemon juice, the effect of the major constituents of lemon juice (citric acid and/or malic acid and/or ascorbic acid) on iron accessibility from hummus were investigated in a new experiment. Therefore, a batch of classical hummus without lemon juice was prepared and is referred to here as “ChpTG” since it contained minimally processed chickpea, tahini, and garlic. The acids were added in solo, dual or triple combinations. Table 4 shows the amount of total iron, the dialyzable iron and the iron bioaccessibility percentage in freeze-dried samples of eight HDA_1→8_ (hummus with different acids). The percentages of iron bioaccessibility are also illustrated in Figure 4.

There is a significant increase in iron bioaccessibility by more than 100% regardless of the nature (citric, malic, and ascorbic acid) and the combination of acids added (in solo, dual or triple combination). Iron bioaccessibility is highest where citric acid is present. The dual combination of citric acid with ascorbic acid (24.21%) or with malic acid (22.61%) promotes iron bioaccessibility more than the triple combination of all the three acids (20.47%). Ascorbic acid has the least increasing effect (14%) in comparison with malic acid (17.15%) or citric acid (19.92%) added individually. The effects of the acids on hummus cannot be well analyzed from the above table; a Pareto chart is inevitable to confirm these results.

### 3.5. Effects of Citric, Malic and Ascorbic Acids on Iron Absorption from Hummus 

**Figure 5:**
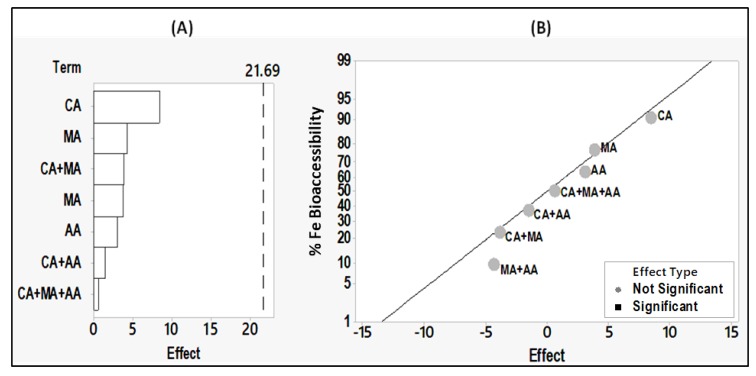
Pareto chart (**A**) and the Normal plot (**B**) of the effects of each acid added alone or in combination, on iron bioaccessibility in the HDA samples according to the 2^3^ experimental design (full factorial, α = 0.05). (HDA: hummus with different acids). CA: citric acid, MA: malic acid, AA: ascorbic acid. Each acid is at 4 mmol/100 g of ChpTG.

For a deeper analysis of the effect of each of the acids added alone or in dual or triple combinations on iron bioaccessibility from hummus, the experimental design 2^3^ (full factorial) was performed and the results are illustrated in Figure 5. There are no significant differences among the effect of all the acids on iron bioaccessibility from hummus, as none of the factors crossed the reference line (21.69, *p*-values are >0.05) (see Figure 5A).

## 4. Discussion

Plant-based food containing iron can help in preventing nutritional iron deficiency only when iron is able to be well absorbed by the body. Hummus is a traditional plant-based dish containing iron, but the bioavailability of iron from this food has not been studied, to the best of our knowledge, since 1967 [37]. In addition, the factors modulating iron bioavailability in hummus have never been analyzed. The principal objective of this research work was to investigate the iron bioavailability from hummus as well as the major factors from its preparation that affect iron bioavailability. The preparation of hummus includes the processing of chickpeas and the addition of tahini, lemon juice, and garlic. 

### 4.1. Total Iron Content in Hummus

The total amount of iron reported in this study for hummus is 4.6 mg/100 g dry weight, which is higher than published results [14,60] ranging between 1.2–2.57 mg/100 g hummus. Different factors could lead to the variation of iron content in hummus, noting mainly: varieties and brands of chickpea and tahini used in the experiments, as well as the way they are processed, which influences the total iron content [61]. For example, the present study showed that the total iron content in the two samples containing chickpea, tahini, and garlic only, which are HDPF_III_ (from the first experiment testing the effect of processing and formulation) and HDA_1_ (from the last experiment testing the effect of acids) is respectively 5 and 2.4 mg per 100 g dry weight of hummus. Although the same batch of chickpeas was used, the processing of chickpeas differed from autoclaving in HDPF (Table 3) to boiling them in HDA (Table 4). This is in conformity with a previous study showing the reduced iron content in boiled chickpeas relatively to autoclaved ones in the same experimental conditions [28]. This could be due to the leaching and loss of more nutrients in the water due to the longer cooking duration of boiling [28].

### 4.2. Lemon Juice, Autoclaving, and Tahini: Principal Modulators

The present study also showed that lemon juice, autoclaving and tahini were the only factors that had a significant effect on iron bioaccessibility from hummus; while lemon juice and autoclaving were shown to be enhancers, tahini was identified as an inhibitor. 

This is the first study showing the promoting effect of lemon juice on hummus. The beneficial effect was expected and is in conformity with previous studies supporting the enhancing effect of lemon juice or its constituents on iron absorption. The reason for this increasing role is commonly attributed to the presence of vitamin C and organic acids that chelate iron to make it more soluble, and reduce it from Fe^3+^ to Fe^2+^, to be available for absorption [43,62]. The acidification also helps the protonation of phytate (phosphate groups, RPO_4_^2−^ + H^+^ ⇆ RPO_4_H^−^ + H^+^ ⇆ RPO_4_H_2_) and could release iron ions in the aqueous phase of hummus. The pKa values of phytic acid are not fully known, but the higher values (over 5) indicate that the protonation begins rapidly when the pH decreases: 9.5; 9.2; 8; 6.25; 5.2; 3.2; 2.4; 1.9 [63].

Among all the processing methods for chickpeas analyzed in this study, autoclaving was the only method to have a significant effect on iron bioaccessibility. The enhancing effect of autoclaving is in conformity with other studies showing that this cooking method significantly increased iron absorption by reducing the antinutritional factors of iron [23,28,64]. 

In contrast to lemon juice and autoclaving, there is scarce information about the inhibiting effect of tahini on iron. Cowan and co-workers (1967) [37] have reported a lower iron bioavailability in tahini alone (67%) than chickpea alone (91%). Yet, while they suggested the probable inhibitory effect of tahini on iron bioavailability in hummus, this present study confirmed it using the in vitro digestion/Caco-2 cell model (Figure 3). The components of tahini responsible for its inhibiting effect on iron bioavailability from hummus were not investigated in this study, but could be attributed to the presence of well-recognized inhibitors of iron absorption in the sesame paste: phytate, oxalate, calcium, and polyphenols [65,66,67,68]. 

Among the three important identified modulators of iron bioavailability: autoclave, tahini, and lemon juice, the latter showed the largest effect (Figure 2). As lemon juice contains three main acids: citric acid, malic acid, and ascorbic acid, their effect on iron bioaccessibility from hummus was further analyzed in order to investigate if the effect of an acid or a certain combination of acids is particularly responsible for the enhancing effect of lemon juice.

### 4.3. Acidic Components of Lemon Juice and Iron Bioaccessibility

The nature and the combinations of the acids did not show any significant effect on iron bioaccessibility from hummus (Figure 5A,B). Although the effect of the acids was not significantly different according to the Pareto chart of the effects, Figure 4 showed that iron bioaccessibility from hummus was higher with citric acid (19.92%) in comparison to malic acid (17.15%), and ascorbic acid (14%). Thereby, citric acid in this study showed a more pronounced effect on iron bioaccessibility than ascorbic acid. This finding is similar to studies investigating iron bioaccessibility [69] but differs from studies investigating iron bioavailability. For example, some in vitro Caco-2 cell studies showed a more enhanced iron uptake with ascorbic acid than with citric acid [43,70]. Other studies including humans revealed a stronger positive correlation of iron absorption, from a rice meal, with ascorbic acid than with citric acid [24] or a significant inhibiting effect of citric acid on iron from a rice and bean-based meal in 49 subjects [71]. 

The results obtained in this experiment are not surprising as the solubility and bioaccessibility of iron were investigated rather than the uptake (a more complex mechanism). One major explanation of the higher iron bioaccessibility obtained with the acids in the following order in this study: citric acid > malic acid > ascorbic acid, is the pKa values of each acid (see Supplementary Information: Table S1). Iron is more soluble when it is bound by chelating agents. The dominant forms of acids dominant at the different pH of the digestion steps depend on the pKa values [72,73,74] (see Supplementary Information: Table S1). As concluded from the latter, citric acid is more dissociated than the other acids along the digestion. It has more negative groups (three carboxylic acid groups) and could, therefore, bind more iron making them more soluble. Moreover, the thermodynamic stability constant reported by Suzuki et al. (1992) [69] of citrate to ferrous iron (4.4) is higher than malic acid (2.6) and ascorbic acid (0.2). The bigger the thermodynamic stability constant is, the better is the binding strength of the chelating agent to iron. This latter prevents iron precipitation and consequently enhances iron bioaccessibility.

Moreover, the results in this study have shown that iron bioaccessibility of hummus including the three combined acids was higher than hummus including lemon juice (20.47% vs. 13.6% from Table 4 and Table 3, respectively). This could be due to the presence of polyphenols and fiber in lemon juice, which decreases the iron bioaccessibility in comparison with the pure form of the three acids used in the laboratory [62]. Moreover, the total content of the three acids added equally (4 mmol/100 g hummus each), could be higher than the total content of the acids found naturally in 14 g of lemon juice added in this study to make hummus; where malic and ascorbic acids content are lower than citric acid content and might be 0.3, 0.02, and 3.5 mmol/100 g hummus [75,76,77] The final pH in hummus could be lower with pure citric acid. However, it cannot be taken into account as the pH of the digesta is controlled by the buffer added during the in vitro digestion for iron dialysis, or during the Caco-2 cell culture.

### 4.4. Iron Bioaccessibility and Iron Bioavailability

This study showed a significant positive correlation between iron bioaccessibility and iron bioavailability when the effects of processing and formulation were investigated including seven different factors (a wide and large range of factors). On the contrary, the results investigating iron bioaccessibility influenced by the acids (a smaller range of factors) did not seem to agree with previous studies done in vivo or on Caco-2 cells. This result is of significance because some studies have shown that iron bioaccessibility did not always correlate with iron bioavailability [42,70,78]. This could be due to the investigation of a small range of factors (e.g., some enhancers [70] or some types of yeasts [42]). Based on this observation, it can be speculated that iron bioaccessibility could be a helpful tool to preselect samples when a large range of factors is being studied (e.g., a food with different ingredients and processing steps). However, when a small range of factors is being investigated, it could be better to start directly with the experiments using the Caco-2 cell model. It must be kept in mind that the more the analytical tool is close to the reality, the more the mechanism of absorption gets more complex and includes several factors such as the physiological pH, temperature, and the various enzymes and signals secreted along the digestion.

### 4.5. Iron Bioaccessibility vs. Effect of Factors on Iron Bioaccessibility

The iron bioaccessibilities from different samples in this study are intercomparable but cannot reveal exactly which factor plays a significant role in iron bioaccessibility. Here comes the interest to employ experimental designs that help to identify the factors that have a significant effect. For example, in the first experiment testing the effect of processing and formulation, the samples containing garlic showed a higher iron bioaccessibility than the samples without garlic (sample HDPF_V_ 13.6% and sample HDPF_IV_ 8.2%, respectively). However, the Pareto chart did not show any significant effect of garlic. Another example in this study is the experiment where iron bioaccessibility from samples of hummus with different combinations of acids were investigated. The iron bioaccessibility among the different samples was shown to be significantly different (Figure 4). Nevertheless, the Pareto chart (Figure 5B) did not reveal any significant difference among the effect of the acids and their different combination on iron bioaccessibility from hummus. Consequently, the comparison of iron bioaccessibility among the different samples is not enough to identify the effectiveness of the factors of interest. Therefore, it is assumed that experimental designs followed by the appropriate analysis tests are indispensable for the identification of the effects of enhancers and inhibitors.

### 4.6. Hummus as a Promising Source of Iron

Hummus contains a relatively higher phytate content than cooked chickpeas [79]. However, this present study showed that iron bioavailability was significantly higher in hummus; it increased by more than 4-fold when both tahini and lemon juice were added to chickpeas (from 7.2 to 30.4 ng of ferritin/mg of total protein in Caco-2 cells, see Figure 3A). In fact, the negative effect of tahini on non-heme iron bioavailability was partially overcome by lemon juice, and the counter-effect of lemon juice on iron inhibitors is supported by in vivo and in vitro studies [41]. Nevertheless, despite the strong increase in iron bioavailability of iron in hummus by adding lemon juice to the chickpea purée, the bioavailability of iron in hummus remains low compared to animal products. The bioavailability of iron in chicken liver, evaluated by the Caco-2 cell model was shown by Pachón et al. (2008) [80] to be 125 ng of ferritin/mg of protein and 85 ng of ferritin/mg of protein with chicken thigh. Not only was iron more bioavailable, but the iron content in animal products was more important: 35 mg of iron/100 g of chicken liver versus 4.7 mg of iron per 100 g of chickpea purée but 3.1 mg/100 g in chicken thigh. On the other hand, iron bioavailability from hummus in this study is better than the iron bioavailability obtained with Pachón et al. (2008) for plant products. Although the final amount of iron given to Caco-2 cells from the hummus (4 µg) in this study was less than the amount of iron given to Caco-2 cells from iron-fortified rice (70 µg) and wheat flour (6 µg) in the study of Pachón et al. (2008), the non-heme iron uptake from hummus was higher than the other plant food. One certain reason behind this outcome is the presence of an iron enhancer (lemon juice) as a main ingredient in the hummus dish, but not in the previously mentioned fortified foods [80]. This comparison encourages the recommendation of the hummus dish as a complementary or an alternative source of iron to iron-fortified foods. This also underlines the importance of focusing on the non-heme iron bioavailability and the factors that modulate it rather than on its content, especially in plant food.

## 5. Conclusions

In conclusion, this present work is the first to investigate the effect of processing and formulation factors on iron bioaccessibility and bioavailability from the hummus dish (complex system). The results obtained show that hummus could be a promising source of iron as the iron bioavailability increased significantly from chickpea alone to when both lemon juice and tahini were added to make hummus. Autoclaving showed a significant positive effect on iron bioaccessibility and is, therefore, encouraged to be chosen over traditional boiling for cooking chickpeas. Clinical studies are needed to investigate further the iron absorption from hummus in humans, and the effect of the combined food consumed concomitantly.

## Figures and Tables

**Table 1:** The screening Hadamard matrix with seven factors and eight samples of hummus with different processing and formulation (HDPF).

	Processing of Chickpeas				Formulation of Hummus		
HDPF Samples	Pre-Cooking	with Bicarbonate	Cooking	Seed Coat	Tahini	Lemon Juice	Garlic
I	+1	+1	+1	−1	+1	−1	−1
II	−1	+1	+1	+1	−1	+1	−1
III	−1	−1	+1	+1	+1	−1	+1
IV	+1	−1	−1	+1	+1	+1	−1
V	−1	+1	−1	−1	+1	+1	+1
VI	+1	−1	+1	−1	−1	+1	+1
VII	+1	+1	−1	+1	−1	−1	+1
VIII	−1	−1	−1	−1	−1	−1	−1
High level +1	48 h Germination	1.4 g	Autoclave 120 °C, 20 min	Removed	21.5 g	14 g	1 g
Low level −1	12 h Soaking	0 g	Boiling for 90 min	Conserved	0 g	0 g	0 g

**Table 2:** The experimental design 2^3^ (full factorial) of hummus with different acid combinations yielding eight samples of hummus with different acid content (HDA).

	Acids Added per 100 g Hummus		
HDA Samples	Citric Acid (4 mmol)	Malic Acid (4 mmol)	Ascorbic Acid (4 mmol)
1	−1	−1	−1
2	+1	−1	−1
3	−1	+1	−1
4	+1	+1	−1
5	−1	−1	+1
6	+1	−1	+1
7	−1	+1	+1
8	+1	+1	+1
High Level +1	Added	Added	Added
Low Level −1	Not added	Not added	Not added

**Table 3:** Total iron (mg/100 g), dialyzable iron (mg/100 g), and accessible iron (%) in the eight samples of hummus with different processing and formulations (HDPF_I__→VIII_).

HDPF Samples	Ingredients Added to Chickpeas	Total Fe (mg/100 g)	Dialyzable Fe (mg/100 g)	Bioaccessibility of Fe (%)
I	Chp + T	4.67 ± 0.43 ^a^	0.19 ± 0.01 ^de^	4.12 ± 0.13 ^de^
II	Chp + LJ	4.39 ± 0.31 ^a^	1.32 ± 0.02 ^a^	30.17 ± 0.51 ^a^
III	Chp + T + G	5.02 ± 0.42 ^a^	0.25 ± 0.01 ^cd^	5.00 ± 0.13 ^d^
IV	Chp + T+ LJ	4.25 ± 0.32 ^a^	0.35 ± 0.01 ^c^	8.23 ± 0.35 ^c^
V	Chp + T + LJ + G	4.60 ± 0.32 ^a^	0.63 ± 0.02 ^b^	13.60 ± 0.39 ^b^
VI	Chp + LJ + G	4.70 ± 0.35 ^a^	1.33 ± 0.06 ^a^	28.44 ± 1.3 ^a^
VII	Chp + G	4.47 ± 0.26 ^a^	0.24 ± 0.01 ^cde^	5.36 ± 0.26 ^d^
VIII	Chp	5.52 ± 0.44 ^a^	0.13 ± 0.01 ^e^	2.44 ± 0.09 ^e^

**Note:** The values are presented as means ± SEM per dry weight (*n* = 3) (SEM: standard error of the mean). Means within the same column that do not share a letter are significantly different (*p* < 0.05), according to the one-way ANOVA test followed by Tukey’s post-hoc test at 95% confidence. Chp: chickpea, T: tahini, LJ: lemon juice, G: Garlic. In the second column, only the ingredients added to chickpea are presented. Table 1 provides further information about the processing of chickpeas.

**Table 4:** Total iron (mg/100 g), dialyzable iron (mg/100 g), and accessible iron (%) in the eight samples of hummus with different acids content (HDA_1__→8_).

HDA Samples	Acids Added to Hummus without Lemon Juice	Total Fe (mg/100 g ChpTG)	Dialyzable Fe (mg/100 g)	Fe Bioaccessibility (%)
1	ChpTG	2.24 ± 0.02	0.10 ± 0.1 ^f^	4.50 ± 0.43 ^f^
2	ChpTG + CA	2.24 ± 0.02	0.42 ± 0.01 ^cd^	18.92 ± 0.30 ^cd^
3	ChpTG + MA	2.24 ± 0.02	0.38 ± 0.0 ^d^	17.15 ± 0.08 ^d^
4	ChpTG + CA + MA	2.24 ± 0.02	0.51 ± 0.02 ^ab^	22.61 ± 0.93 ^ab^
5	ChpTG + AA	2.24 ± 0.02	0.31 ± 0.02 ^e^	14.02 ± 0.67 ^e^
6	ChpTG + CA + AA	2.24 ± 0.02	0.54 ± 0.01 ^a^	24.21 ± 0.66 ^a^
7	ChpTG + AA + MA	2.24 ± 0.02	0.38 ± 0.01 ^d^	16.79 ± 0.29 ^d^
8	ChpTG + CA + MA + AA	2.24 ± 0.02	0.46 ± 0.0 ^bc^	20.47 ± 0.08 ^bc^

**Note:** The values are presented as means ± SEM per dry weight (*n* = 3). Means within the same column that do not share a letter are significantly different (*p* < 0.05), according to the one-way ANOVA test followed by Tukey’s post-hoc test at 95% confidence. ChpTG is a classical hummus without lemon juice (ChpTG = Chp: chickpea + T: tahini + G: garlic). CA: citric acid, MA: malic acid, AA: ascorbic acid. Each acid is at 4 mmol/100 g of ChpTG.

## Supplementary Materials

The following are available online at https://www.mdpi.com/2304-8158/9/4/474/s1, Figure S1: Scheme of iron dialysis from digesta to buffer solution through a dialysis membrane. Figure S2: Scheme of the dual chamber system of iron uptake by the Caco-2 cells. Figure S3: Representation of Pearson’s Correlation critical values’ table. Table S1: Theoretical predominant form(s) of acids along the in vitro digestion at different pH values (2, 7, and 8.5).
