# Supplementary material for: Lemon Juice, Sesame Paste, and Autoclaving Influence Iron Bioavailability of Hummus: Assessment by an In Vitro Digestion/Caco-2 Cell Model
Source: Foods. 2020 Apr 10;9(4):474. doi: 10.3390/foods9040474 (PMC7230787; doi:10.3390/foods9040474)
Supplement: Supplementary file 1 [file foods-09-00474-s001.pdf]

Supplementary Materials

Lemon Juice, Sesame Paste, and Autoclaving Influence Iron Bioavailability of Hummus: Assessment by an In Vitro Digestion/Caco-2 Cell Model

Nour Doumani*, Isabelle Severin, Laurence Dahbi, Elias Bou-Maroun, Maya Tueni, Nicolas Sok, Marie-Christine Chagnon, Jacqueline Maalouly and Philippe Cayot

Table of contents

Figure S1. Scheme of iron dialysis from digestat to buffer solution through a dialysis membrane 2

Figure S2. Scheme of the dual chamber system of iron uptake by the Caco-2 cells........ 2

Figure S3. Representation of Pearson's Correlation critical values' table. 3

Table S1. Theoretical predominant form(s) of acids along the *in vitro* digestion at different pH values (2, 7, 8.5)........ 4

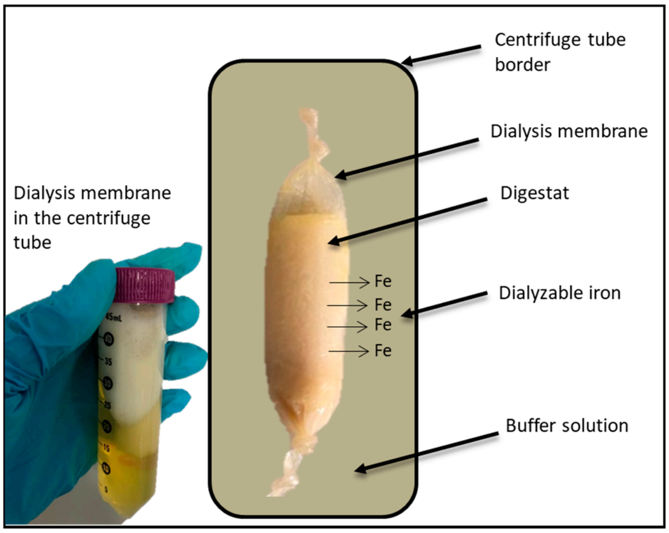

Figure S1. Scheme of iron dialysis from digestat to buffer solution through a dialysis membrane

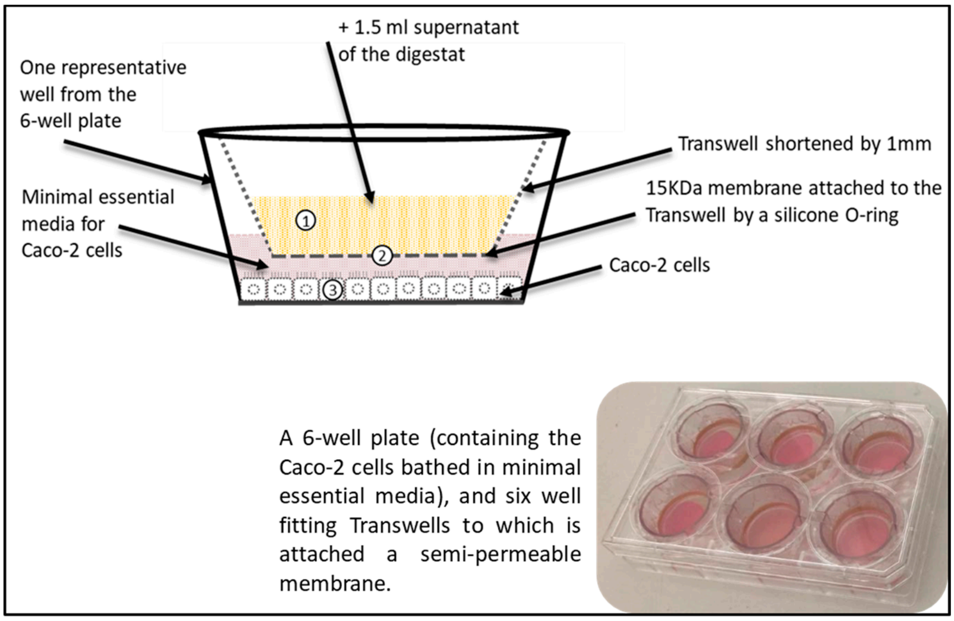

Figure S2. Scheme of the dual chamber system of iron uptake by the Caco-2 cells. 1) Iron in the digestapoured in the upper chamber formed by the Transwell, 2) iron diffusion to the lower chamber through a 15 KDa membrane attached to the Transwell by a silicone O-ring and 3) iron uptake by the Caco-2 cells, and eventually ferritin formation.

Table R Critical Values of the Correlation Coefficient

	Levels of Significance for a One-Tailed Test			
	.05	.025	.01	.005
	Levels of Significance for a Two-Tailed Test			
df	.10	.05	.02	.01
1	.988	.997	.9995	.9999
2	.900	.950	.980	.990
3	.805	.878	.934	.959
4	.729	.811	.882	.917
5	.669	.754	.833	.874
6	.622	.707	.789	.834
7	.582	.666	.750	.798
8	.549	.632	.716	.765
9	.521	.602	.685	.735
10	.497	.576	.658	.708
11	.476	.553	.634	.684
12	.458	.532	.612	.661
13	.441	.514	.592	.641
14	.426	.497	.574	.623
15	.412	.482	.558	.606
16	.400	.468	.542	.590
17	.389	.456	.528	.575
18	.378	.444	.516	.561
19	.369	.433	.503	.549
20	.360	.423	.492	.537
21	.352	.413	.482	.526
22	.344	.404	.472	.515
23	.337	.396	.462	.505
24	.330	.388	.453	.496
25	.323	.381	.445	.487
26	.317	.374	.437	.479
27	.311	.367	.430	.471
28	.306	.361	.423	.463
29	.301	.355	.416	.456
30	.296	.349	.409	.449
32	.287	.339	.397	.436
34	.279	.329	.386	.424
36	.271	.320	.376	.413
38	.264	.312	.367	.403
40	.257	.304	.358	.393
42	.251	.297	.350	.384

(continued)

Figure S3. Representation of Pearson's Correlation critical values' table [59].

Table S1. Theoretical predominant form(s) of acids along the in vitro digestion at different pH values (2, 7, 8.5).

	pH 2 (gastric phase)	pH 7 (intestinal phase)	pH 8.5 (end of intestinal phase)
**Citric acid[72]**<br>**pKa1 3.1**<br>**pKa2 4.7**<br>**pKa3 6.4**	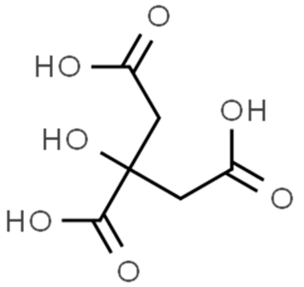<br>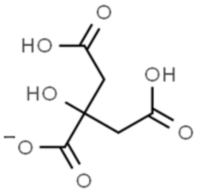	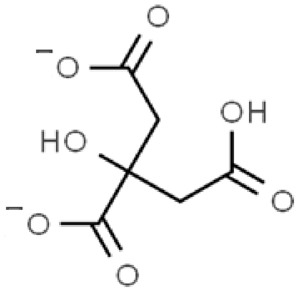	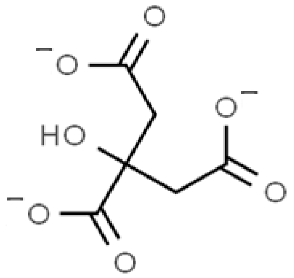
**Malic acid[73]**<br>**pKa1 3.51**<br>**pKa2 5.03**	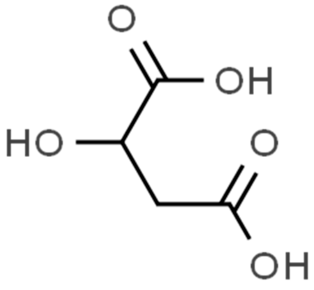<br>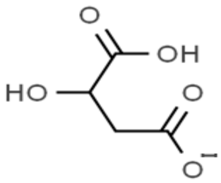	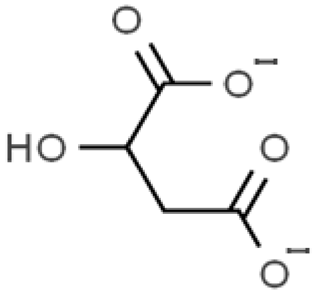	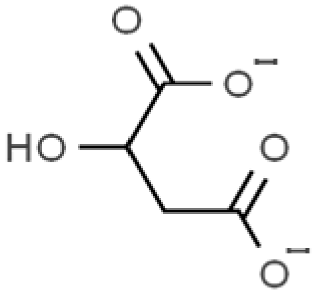
**Ascorbic acid[74]**<br>**pKa1 4.17**<br>**pKa2 11.57**	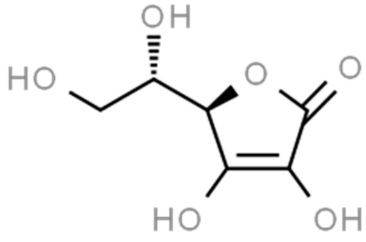	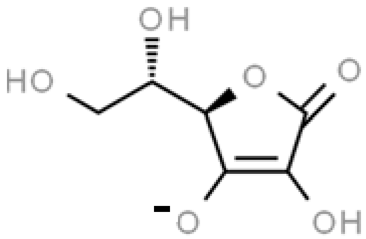	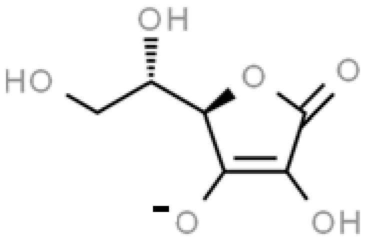
